# Case Report: A potentially pathogenic new variant of the *REN* gene found in a family experiencing autosomal dominant tubulointerstitial kidney disease

**DOI:** 10.3389/fped.2024.1415064

**Published:** 2024-12-11

**Authors:** Jingyu Ma, Zhijuan Hu, Qiong Liu, Jing Li, Jiejie Li

**Affiliations:** ^1^ Department of Internal Medicine, North China University of Science and Technology, Tangshan, China; ^2^ Department of Nephrology, Hebei General Hospital, Shijiazhuang, China; ^3^ Department of Internal Medicine, Hebei Medical University, Shijiazhuang, China

**Keywords:** ADTKD, acute kidney injury, hyperuricemia, *REN*, renal insufficiency

## Abstract

**Background:**

Autosomal dominant tubulointerstitial kidney disease (ADTKD) caused by *REN*-causing pathogenic variants (ADTKD-*REN*) is a rare group of heritable diseases. ADTKD-*REN* often manifests in childhood with symptoms such as mild hypotension, chronic kidney disease, hyperkalemia, anemia, and acidosis. The diagnosis of ADTKD-*REN* remains challenging.

**Case presentation:**

We describe a 14-year-old boy with acute kidney injury who was found to have a heterozygous missense mutant c.1085G>A;p.Cys362Tyr (not previously reported in the literature) through Sanger sequencing genetic testing. This confirmed a genetic disorder with a probable autosomal dominant inheritance pattern. Notably, despite a family history of autosomal dominant polycystic kidney disease, he was diagnosed with ADTKD.

**Conclusion:**

This case identified a new variant in the *REN* gene, expanding the known spectrum of *REN* pathogenic variants. In addition, the importance of family history and genetic testing in confirming the diagnosis is emphasized. Genetic sequencing should be pursued when there are indications for testing.

## 1 Introduction

The prevalence of chronic kidney disease (CKD) has risen significantly in recent years, with an increasing number of patients requiring renal replacement therapy (1). The prevention and treatment of CKD have become significant public health challenges worldwide. Research has extensively focused on primary kidney disease, diabetic nephropathy, and hypertensive kidney damage. However, with the progress of genetic technology, the proportion of gene nephropathy in the etiology of end-stage renal disease (ESRD) has also gradually increased. Autosomal dominant tubulointerstitial kidney disease (ADTKD) is a rare hereditary disorder characterized by progressive loss of kidney function, non-significant urinalysis, and tubulointerstitial fibrosis.

The 2015 Kidney Disease: Improving Global Outcomes (KDIGO) guideline systematically addresses autosomal dominant tubulointerstitial nephropathy (2); five ADTKD-causing genes (*MUC1, UMOD, REN, HNF1B*, and *SEC61A1*) and their corresponding genotypes have been identified (3). These genes encode various proteins with renal and extra-renal functions, resulting in distinct clinical manifestations. Among these, pathogenic variants in *UMOD*, *MUC1*, and *REN* are the most prevalent causes of ADTKD. Renin synthesis is a critical step in the renin–angiotensin system (RAS), and in ADTKD-*REN*, heterozygous *REN* pathogenic variants lead to reduced renin production. This can result in mild hyperkalemia, anemia, acidemia, and a predisposition to acute kidney injury (4).

In this case report, a 14-year-old male adolescent was genetically tested and found to have a heterozygous missense variant in the *REN* gene (c.1085G>A; p.Cys362Tyr), a pathogenic variant not previously documented in the literature. The discovery enriches the genetic database of ADTKD and further suggests that genetic testing is the most effective diagnostic tool.

## 2 Case presentation

A 14-year-old boy presented with intermittent fever for over a month, elevated blood creatinine for 10 days, and abdominal pain for 3 days. Laboratory tests revealed an influenza A virus infection and right lung pneumonia confirmed by chest CT. Kidney function showed a creatinine level of 137.7 μmol/L (1.56 mg/dl) and uric acid of 732 μmol/L (12.2 mg/dl). The patient was treated with oseltamivir and ceftriaxone sodium on an outpatient basis with no improvement in symptoms. Therefore, he came to our hospital.

**Figure 1:**
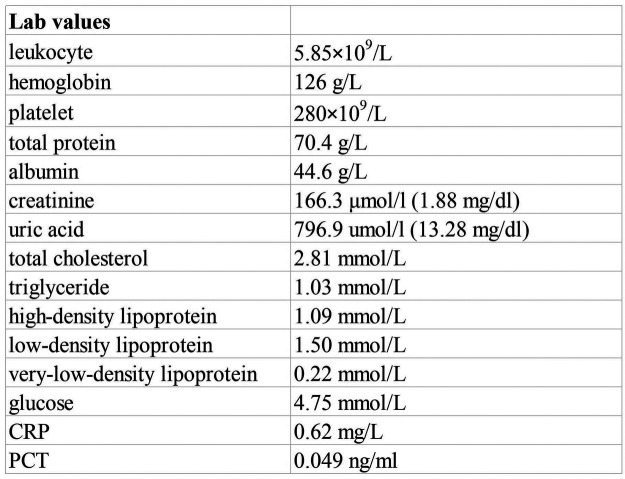
Laboratory data.

The physical examination was unremarkable. Laboratory data showed total protein of 70.4 g/L, albumin of 44.6 g/L, creatinine of 166.3 μmol/L (1.88 mg/dl), uric acid of 796.9 μmol/L (13.28 mg/dl), blood potassium of 4.7 mmol/L, carbon dioxide combining power of 20.2 mmol/L, and estimated glomerular filtration rate (eGFR) of 56.02 ml/min/1.73 m^2^ (Figure 1). Normal or negative findings in routine urinalysis, serum complement, antinuclear antibodies (ANA), anti-double-stranded DNA (anti-dsDNA), antineutrophil cytoplasmic antibodies (ANCA), rheumatoid factor, T-cell testing for tuberculosis infection, and blood sedimentation rate. A kidney ultrasound revealed slightly enhanced echogenicity in the parenchyma of both kidneys and disorganized sinusoidal structures. A cardiac ultrasound suggested a bicuspid aortic valve with increased supravalvular flow velocity and low to moderate regurgitation. An abdominal ultrasound showed multiple gallbladder stones with no apparent abnormalities in the liver, pancreas, and spleen. Due to the sustained elevation of serum creatinine levels from baseline, we considered the patient to be experiencing acute kidney injury. Notably, he had a family history of polycystic kidneys.

**Figure 2:**
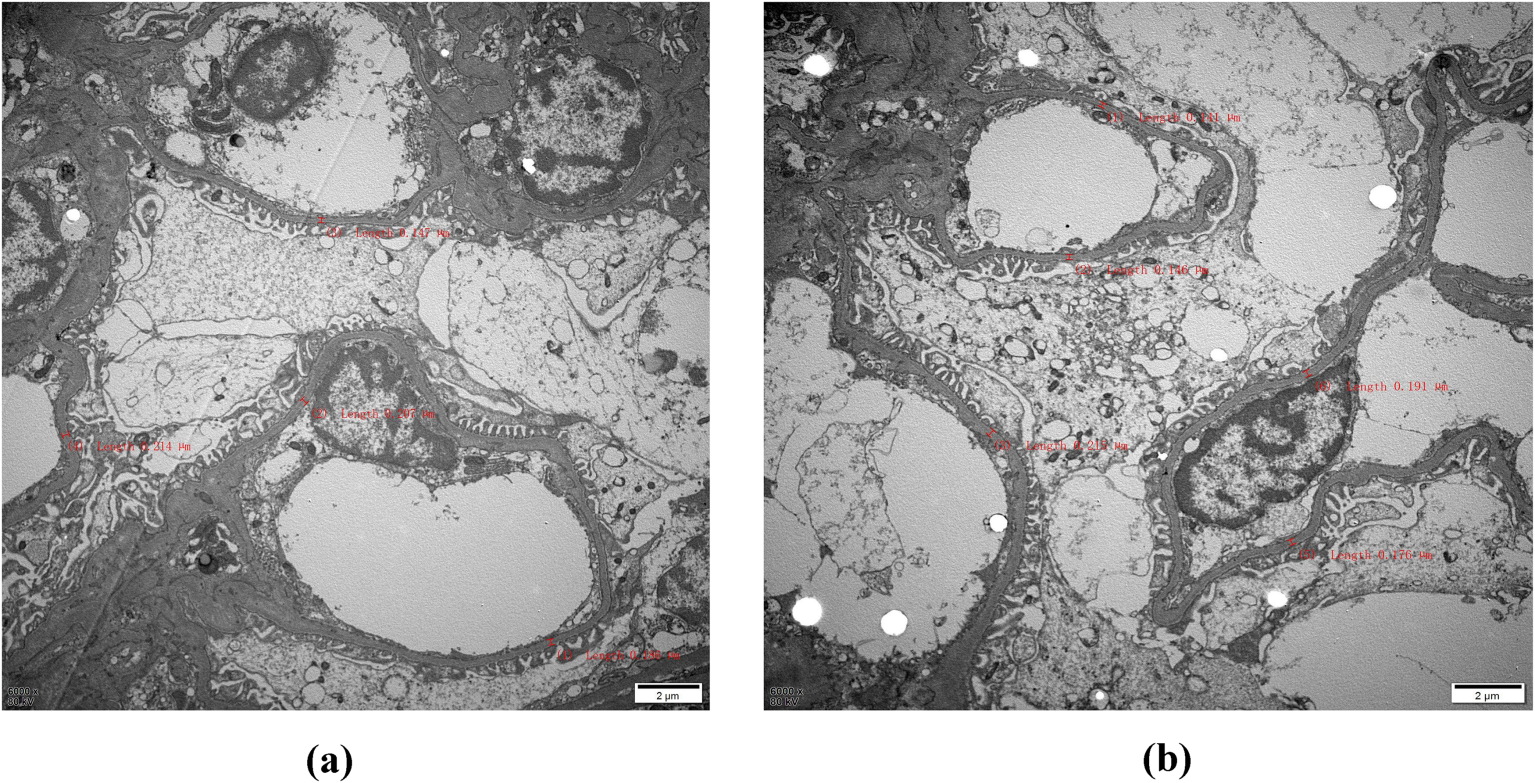
Electron microscopy: The red markings on **(a)** and **(b)** indicate that the thickness of the measured substrate film is less than 250 nm, with most samples being classified as thin. Therefore, it is imperative to conduct further investigations into thin basement membrane disease and Alport syndrome (magnification, ×6,000).

**Figure 3:**
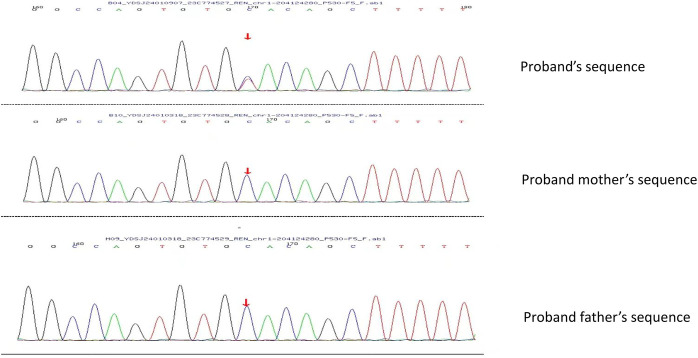
Using Sanger sequencing, it was verified that the bases shown in the peak graph were reverse complementary sequences of the base being tested. The proband had a missense pathogenic variant at nucleotide 1,085 from guanine G to adenine A, which resulted in amino acid 362 being changed from Cys to Tyr. No pathogenic variant was seen in the proband's father and mother at this locus.

**Figure 4:**
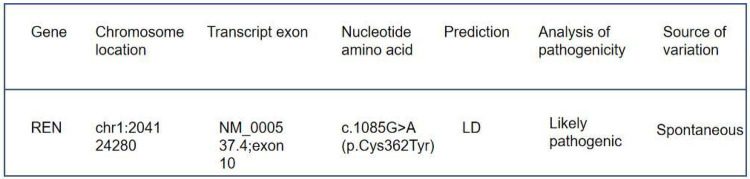
Genetic test results: the detected gene was *REN*, the pathogenic variant was heterozygous, the origin of the pathogenic variant was spontaneous, and the pathogenic variant was judged to be a suspected pathogenic variant according to American College of Medical Genetics and Genomics (ACMG) guidelines.

Immunofluorescence microscopy revealed negative staining IgA, IgG, IgM, C3, C1q, Fibrin, HbcAg, HbsAg, Kappa, and Lambda. A light microscopic examination of the renal specimen, containing 33 glomeruli, showed global sclerosis in 12 glomeruli and segmental sclerosis in 1 glomerulus. The remaining glomeruli exhibited no remarkable lesions. There was 5% tubular atrophy with few protein casts and red blood cell casts. No interstitial fibrosis was observed, and the renal arterioles appeared normal. Electron microscopy revealed marked vacuolar degeneration of the capillary endothelial cells. The basement membrane was predominantly thin, with a thickness of less than 250 nm, and exhibited segmental effacement of the podocyte foot process. No electron-dense deposits were observed. The findings could not exclude thin basement membrane nephropathy or Alport syndrome (Figure 2). Clinical data and blood samples were collected from the patient and his family for genetic testing using high-throughput sequencing and Sanger sequencing, respectively (Figure 3). A heterozygous missense variant (c.1085G>A;p.Cys362Tyr) was found in the *REN* gene, and a spontaneous heterozygous missense pathogenic variant was identified in the patient (Figure 4).

The patient was treated with intravenous methylprednisolone at 40 mg for 10 days, followed by a reduction in dose at 20 mg for 6 days. His renal function improved, with his creatinine levels decreasing to 100.3 μmol/L (1.13 mg/dl) and uric acid levels to 374.5 μmol/L (6.2 mg/dl). He was discharged from hospital on 16 mg of oral methylprednisolone, which was tapered off in the outpatient clinic.

## 3 Discussion

ADTKD was first introduced in 1945 by Smith and Graham (5). It was originally named medullary cystic nephropathy based on imaging owing to a limited understanding of genetic diseases at the time. Currently, ADTKD, autosomal dominant polycystic kidney disease (ADPKD), and Alport syndrome are considered the three major hereditary kidney diseases (6). Clinical manifestations of ADTKD tend to be non-specific and may not be evident on urinary examination and renal ultrasonography, yet the disease progresses slowly and inevitably to end-stage renal disease. ADTKD is associated with at least five pathogenic variants: *UMOD, MUC1, REN, HNF1B*, and the rare *SEC61A1*. As of the end of November 2022, 35 reports of pathogenic variants in the *REN* gene are documented in the Global Variome shared LOVD database, with 16 identified disease-causing variants (7).

The *REN* gene encodes prorenin, which is hydrolyzed to form renin, a key enzyme involved in the breakdown of angiotensinogen (8). Pathogenic variants in the *REN* gene lead to the production of aberrant renin that accumulates in cells, inducing apoptosis. These pathogenic variants manifest in two ways: the more common childhood or adolescent onset, often associated with acute kidney injury, anemia, gout, and hyperkalemia, and the adult onset, which resembles CKD (9). In this paper, we report the case of a male adolescent with acute kidney injury. We considered various factors, including primary kidney disease, drugs, infection, and metabolism disturbance. Combined with his family history, we speculated he might also have a polycystic kidney, prompting us to conduct a genetic examination. The result was surprising: despite a family history of polycystic kidney disease, the patient was confirmed to have ADTKD with a previously unreported pathogenic variant site. It remains unclear whether this missense pathogenic variant is entirely independent.

In summary, the patient presented with hyperuricemia and acute kidney injury at the age of 14 years. His urinary sediment was bland, and there was no hyperkalemia, anemia, or acidosis. There were no uric acid kidney stones present, suggesting that the patient did not have an overproduction of uric acid. Genetic testing revealed a p.Cys362Tyr variant in the *REN* gene. Neither of his parents had this variant. The renal biopsy revealed glomerulosclerosis in 12/33 glomeruli, with limited tubular atrophy and tubulointerstitial fibrosis. After treatment for acute kidney injury, the patient had baseline mild chronic kidney disease, with serum creatinine of 1.13 mg/dl. Based on the patient's clinical presentation, laboratory findings, and the results of the renal biopsy, we diagnosed the patient with chronic kidney disease. The viral infection resulted in AKI, which was identified as the causative factor for the patient's renal insufficiency rather than an etiological one. Consequently, we promptly withdrew hormone therapy at a later stage.

The finding of a new genetic variant in the *REN* gene (p.Cys362Tyr) requires further investigation to determine if this variant is causative or simply a non-pathogenic variant. *In silico* (computer-generated) scores can be calculated to determine if this amino acid change would result in a likely pathogenic or non-pathogenic pathogenic variant. In this case, the variant has a Rare Exome Variant Ensemble Learner (REVEL) score of 0.74, which is supportive of the variant being deleterious. The variant was not found in any other individuals in the gnomAD database, which is also supportive. In addition, the cysteine residue at 362 forms a disulfide bond with the cysteine residue at 325. A family has been reported with a similar phenotype and a pathogenic variant at p.Cys325Arg (11). Thus, in silico evidence would suggest that the pathogenic variant is causative. One must then compare the clinical findings in other patients with similar pathogenic changes. Rampoldi was the first to describe a pathogenic variant in the *REN* gene (p.L381P) as a cause of ADTKD. Živná et al. then described 15 individuals with pathogenic variants in the mature *REN* gene. All patients reported with *REN* pathogenic variants have presented after the age of 20 years, often with gout. They have experienced slowly progressive chronic kidney disease, leading to end-stage kidney disease at a mean age of 64 years (10). The patients have a bland urinary sediment and no hematuria.

Thus, there is strong evidence to suggest that the patient has ADTKD-*REN*. He has chronic kidney disease, bland urinary sediment, and hyperuricemia. An *in silico* examination suggests that the *REN* variant is causative. The patient presented earlier than other patients with a pathogenic variant in the mature *REN* variant. It is likely that the patient developed AKI from his viral illness and this led to the detection of his baseline mild chronic kidney disease. It is likely that the *REN* pathogenic variant predisposed him to acute kidney injury.

This case illustrates the importance of genetic testing in making a genetic diagnosis (11). Patients with pathogenic variants in the mature *REN* peptide, in general, have a good prognosis and only require symptomatic treatment (12). The patient should have close follow-up, though the literature would suggest that he should not require renal replacement therapy for many years.

## 4 Conclusions

The discovery of this case further expands the ADTKD-*REN* pathogenic variant database with a new missense pathogenic variant type. In conclusion, the clinical presentation of ADTKD is often unremarkable, leading to potential misdiagnosis. Nephrologists are encouraged to take a thorough patient history when suspecting the disease and to perform gene sequencing early.

## Data Availability

The datasets generated and analyzed in this case report are not publicly available due to privacy and ethical concerns, as the study involves a single patient. Requests to access the datasets can be directed to the corresponding author, Zhijuan Hu, at huzhijuan1972@163.com.
